# Immunoregulatory Actions of Epithelial Cell PPAR γ at the Colonic Mucosa of Mice with Experimental Inflammatory Bowel Disease

**DOI:** 10.1371/journal.pone.0010215

**Published:** 2010-04-20

**Authors:** Saroj K. Mohapatra, Amir J. Guri, Montse Climent, Cristina Vives, Adria Carbo, William T. Horne, Raquel Hontecillas, Josep Bassaganya-Riera

**Affiliations:** Nutritional Immunology and Molecular Nutrition Laboratory, Virginia Bioinformatics Institute, Virginia Polytechnic Institute and State University, Blacksburg, Virginia, United States of America; Charité-Universitätsmedizin Berlin, Germany

## Abstract

**Background:**

Peroxisome proliferator-activated receptors are nuclear receptors highly expressed in intestinal epithelial cells (IEC) and immune cells within the gut mucosa and are implicated in modulating inflammation and immune responses. The objective of this study was to investigate the effect of targeted deletion of PPAR γ in IEC on progression of experimental inflammatory bowel disease (IBD).

**Methodology/Principal Findings:**

In the first phase, PPAR γ flfl; Villin Cre- (VC-) and PPAR γ flfl; Villin Cre+ (VC+) mice in a mixed FVB/C57BL/6 background were challenged with 2.5% dextran sodium sulfate (DSS) in drinking water for 0, 2, or 7 days. VC+ mice express a transgenic recombinase under the control of the Villin-Cre promoter that causes an IEC-specific deletion of PPAR γ. In the second phase, we generated VC- and VC+ mice in a C57BL/6 background that were challenged with 2.5% DSS. Mice were scored on disease severity both clinically and histopathologically. Flow cytometry was used to phenotypically characterize lymphocyte and macrophage populations in blood, spleen and mesenteric lymph nodes. Global gene expression analysis was profiled using Affymetrix microarrays. The IEC-specific deficiency of PPAR γ in mice with a mixed background worsened colonic inflammatory lesions, but had no effect on disease activity (DAI) or weight loss. In contrast, the IEC-specific PPAR γ null mice in C57BL/6 background exhibited more severe inflammatory lesions, DAI and weight loss in comparison to their littermates expressing PPAR γ in IEC. Global gene expression profiling revealed significantly down-regulated expression of lysosomal pathway genes and flow cytometry results demonstrated suppressed production of IL-10 by CD4+ T cells in mesenteric lymph nodes (MLN) of IEC-specific PPAR γ null mice.

**Conclusions/Significance:**

Our results demonstrate that adequate expression of PPAR γ in IEC is required for the regulation of mucosal immune responses and prevention of experimental IBD, possibly by modulation of lysosomal and antigen presentation pathways.

## Introduction

Inflammatory bowel disease (IBD), with its two clinical manifestations Crohn's Disease (CD) and Ulcerative Colitis (UC), is a chronic gastrointestinal disorder associated with disruption of the balance between gut commensal bacteria and host responses at the mucosa. The mucosal barrier consists of epithelial tight junctions regulated by cytokines and the underlying immune cell network. Damage to the mucosal barrier is considered sufficient for causing intestinal inflammation. On the other hand, commensal bacteria dampen inflammation via nucleocytoplasmic redistribution of peroxisome proliferator-activated receptor (PPAR) γ and RelA subunit of transcription factor NF-κB [1]. A working model of IBD starts with alterations of the epithelial barrier followed by innate immune responses against the gut microbiota. Later changes involving lymphocytes drive the tissue damage associated with the disease [1].

PPAR γ, a member of the nuclear receptor group of transcription factors, not only regulates lipid and carbohydrate metabolism, but has been recognized as playing an important role in the immune response through its ability to down-modulate the expression of inflammatory cytokines and to direct immune cell differentiation towards anti-inflammatory phenotypes [2], [3]. PPAR γ is highly expressed in the intestinal epithelium, immune cells and adipocytes, and regulates a number of genes participating in metabolism, proliferation, signal transduction, and cellular motility [4].

In an experimental model of IBD, activation of PPAR γ by conjugated linoleic acid, abscisic acid or other agonists suppresses gut inflammatory lesions, weight loss and inflammatory mediator expression [4], [5], [6], [7], [8]. Most notably, the PPAR γ agonist rosiglitazone showed therapeutic efficacy in humans with UC [9], [10]. However, rosiglitazone and other drugs belonging to the thiazolidinedione (TZD) class of anti-diabetic drugs are unlikely to be adopted for the treatment of IBD because of their significant side effects (i.e., fluid retention, hepatotoxicity, weight gain and congestive heart failure) and a U.S. Food and Drug Administration (FDA)-mandated “black box warning” for rosiglitazone and pioglitazone. Thus, understanding the role of PPAR γ in each cell type involved in the pathogenesis of IBD is critical for the informed development of novel, safer and more efficacious therapeutic and prophylactic agents against IBD. We have previously used a cre-lox recombination system to characterize the immune modulatory actions of PPAR γ in mice with a targeted deletion in both immune and epithelial cells (i.e., MMTV-Cre) [3], [8] or T cells (i.e., CD4-Cre) [11]. Others have shown that mice lacking PPAR γ in the colonic epithelium displayed increased susceptibility to dextran sodium sulfate (DSS)-induced experimental IBD, histological lesions and elevated levels of the pro-inflammatory cytokines IL-6, IL-1β, TNF-α [12]. The objective of this study was to use a systems approach for investigating the underlying mechanisms by which the deletion of PPAR γ in IEC modulates the severity of experimental IBD, immune cell distribution and global gene expression.

## Results

### Effect of the deficiency of PPAR γ ****in IEC and mouse strain on susceptibility to DSS-induced colitis

**Figure 1:**
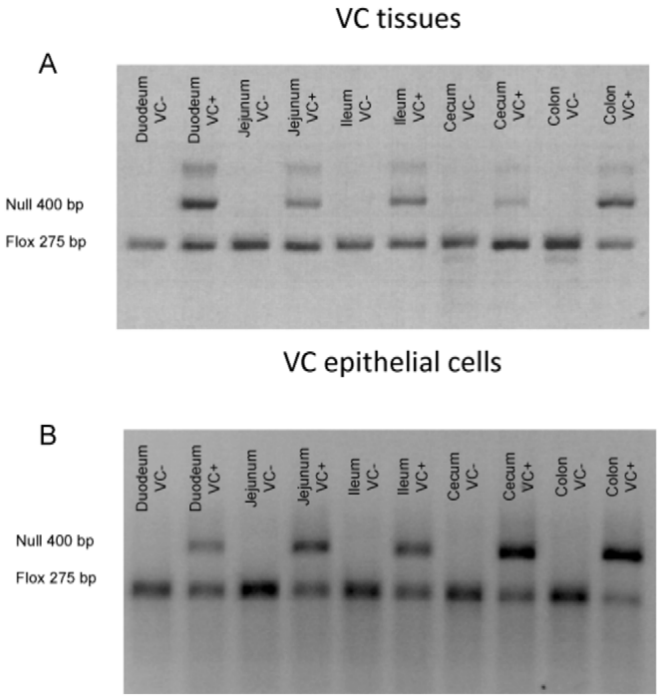
Genotyping of PPAR γ flfl; Villin Cre+ (VC+) and Villin Cre- (VC-) control mice. Conditional deletion of the PPAR γ gene via Villin Cre-mediated recombination was examined in mouse intestine by PCR analysis. The floxed (fl) allele at 275 bp and the null allele at 400 bp. (A) Left to right: depicts fl/fl in homogenized whole duodenum, jejunum, ileum, cecum and colon without recombination (VC-) (*lanes 1, 3, 5, 7 and 9*) or with recombination (VC+) (*lanes 2, 4, 6, 8 and 10*). (B) Left to right: depicts fl/fl in epithelial cells isolated from duodenum, jejunum, ileum, cecum and colon without recombination (VC-) (*lanes 1, 3, 5, 7 and 9*) or with recombination (VC+) (*lanes 2, 4, 6, 8 and 10*).

**Figure 2:**
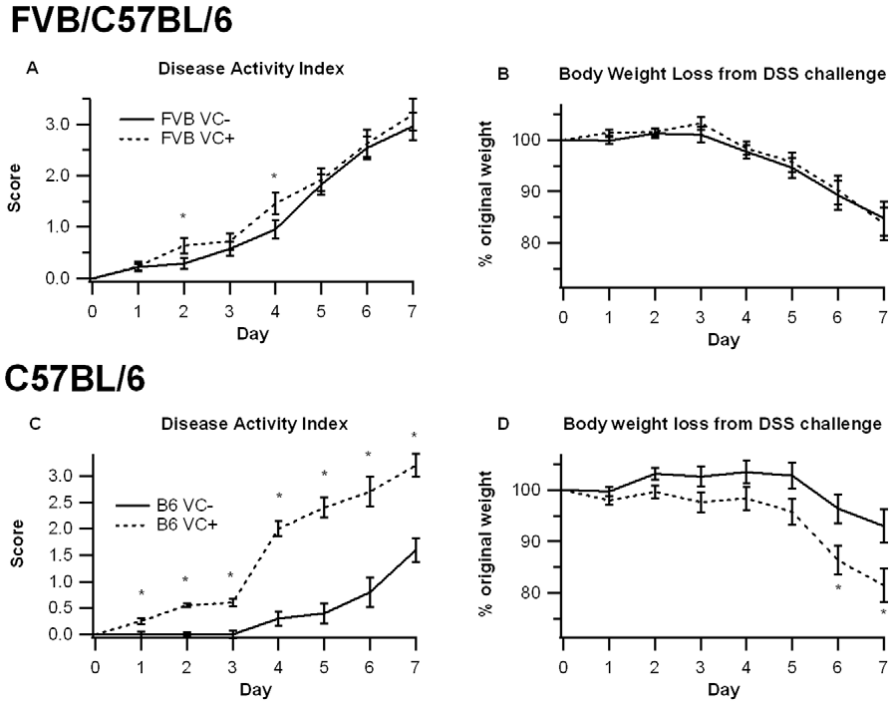
Effect of epithelial cell-specific PPAR γ deletion on disease severity. PPAR γ flfl; Villin Cre+ (VC+) or PPAR γ flfl; Villin Cre- (VC-) mice in a mixed FVB/C57BL/6J (FVB) or C57BL/6J (B6) background were treated with 2.5% dextran sodium sulfate (DSS) or water (no DSS) for 7 days. The disease activity index (DAI), a composite score reflecting clinical signs of the disease (i.e. perianal soiling, rectal bleeding, diarrhea, and piloerection) was assessed daily (A and C) and the average daily loss in body weights (B and D) throughout the 7-day DSS challenge was calculated. Data are represented as mean ± standard error. Points with an asterisk are significantly different (*P*<0.05).

To examine the affect of IEC-specific PPAR γ deficiency and mouse strain on colitis severity VC+ and VC- mice in a mixed FVB/C57BL/6 background were treated with 2.5% DSS for 0, 2, or 7 days. Figure 1A illustrates the deletion of PPAR γ in the duodenum, jejunum, ileum, cecum and colon of VC+ mice as well as in IEC isolated from these tissues (Figure 1B). The results show that the deletion is more efficient in the large intestine (i.e., cecum and colon), compared to small intestine. Unlike findings reported by Adachi and colleagues, we found no significant differences in disease activity (DAI) or body weight loss between groups (Figure 2A-B). However, the targeted deletion of IEC PPAR γ in mice with a C57BL/6 background resulted in significant weight loss and disease activity in comparison to mice expressing PPAR γ in IEC (Figures 2C-D).

**Figure 3:**
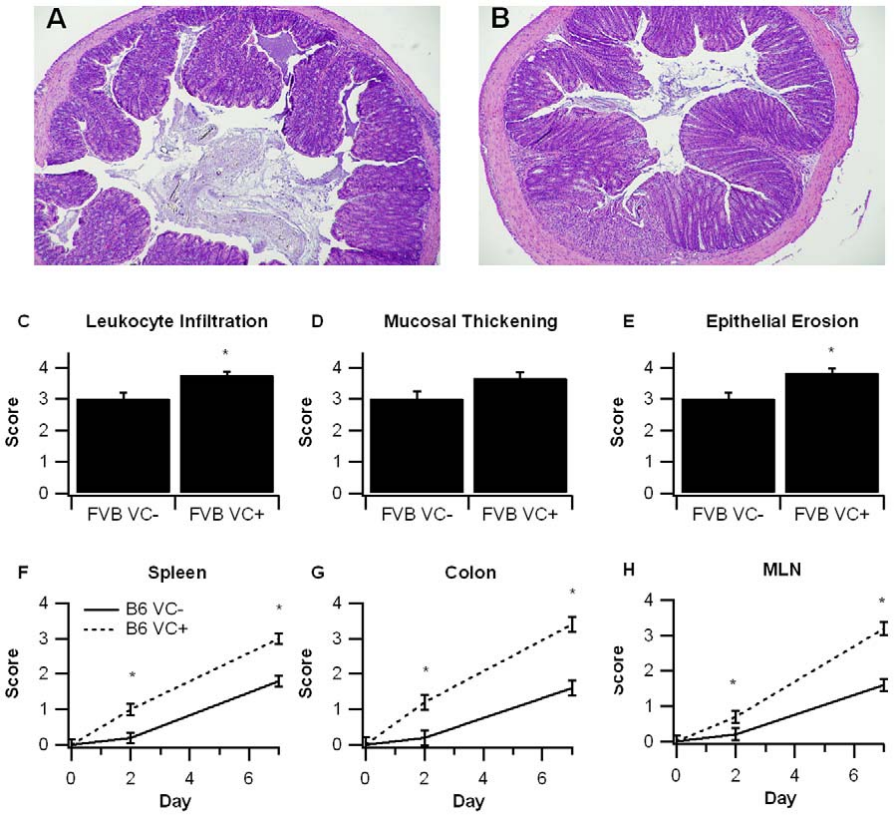
Effect of intestinal epithelial cell-specific PPAR γ deletion on colon histopathology and inflammation. PPAR γ flfl; Villin Cre+ (VC+) or PPAR γ flfl; Villin Cre- (VC-) mice with a mixed FVB/C57BL6/J (FVB) or C57BL6/J (B6) background were treated with 2.5% dextran sodium sulfate (DSS) or water (no DSS) for 7 days. Representative photomicrographs of colonic samples from VC- (A) and VC+ (B) FVB mice with DSS colitis (Original magnification, 40×). Colonic specimens from FVB mice underwent blinded histological examination and were scored (1–4) on leukocyte infiltration (C), and mucosal wall thickening (D), and epithelial erosion (E) on day 7 of the challenge. In B6 mice spleen (F), colon (G), and mesenteric lymph nodes MLN (H) were scored based on macroscopic signs of inflammation on days 2 and 7. Data are represented as mean ± standard error. Points with an asterisk are significantly different (*P*<0.05).

Despite there being no significant differences in either disease activity or body weight between FVB VC- and FVB VC+ mice, there were significant differences observed at the histopathological level. FVB VC+ mice had significantly greater leukocyte infiltration, and erosion of the mucosal epithelium was significantly worsened in comparison to the control VC- mice (Figure 3). VC+ mice with a pure C57BL/6 background showed significantly greater signs of macroscopic inflammation in the spleen, colon, and MLN than VC- mice (Figures 3F-H). In line with the DAI the enhanced inflammation was evident at day 2 and was significantly exacerbated at day 7 of the DSS challenge.

### Mice expressing PPAR γ in IEC have greater percentages of CD4+IL10+ T cells in MLN

**Figure 4:**
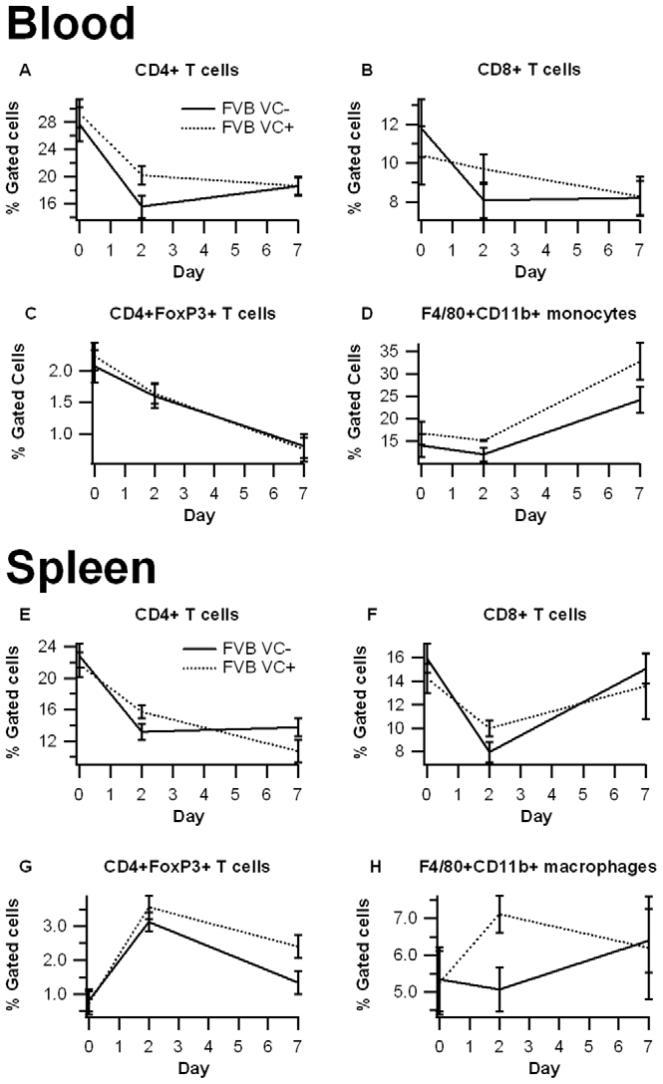
Effect of epithelial cell-specific PPAR γ deletion on immune cell subsets in blood and spleen in FVB/C57BL/6J mice. Blood (A–D) and spleen (E–H) from PPAR γ flfl; Villin Cre+ (VC+) or PPAR γ flfl; Villin Cre- (VC-) mice with a mixed FVB/C57BL/6J background (FVB) were immunophenotyped. Data were collected on days 0, 2, and 7 of DSS challenge and were analyzed with FACS Diva software. Data are represented as mean ± standard error. There were no statically significant differences between groups (*P*<0.05).

To assess whether the targeted deficiency of IEC PPAR γinfluences the phenotype of immune cells we performed flow cytometric analyses on cells isolated from the spleen, blood, and MLN. Our analysis indicated that the deficiency of IEC PPAR γ in FVB mice had no significant impact on the percent of CD4+ or CD8+ T cells in any of the tissues analyzed (Figure 4). There was a numerical trend towards increased F4/80+CD11b+ macrophages/monocytes in the spleen and blood on days 2 and 7, respectively, though these data were not statistically significant.

**Figure 5:**
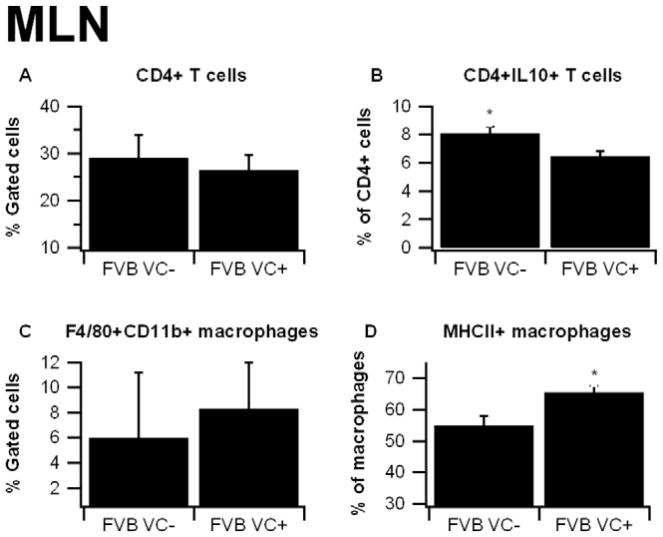
Effect of epithelial cell-specific PPAR γ deletion on immune cell subsets in mesenteric lymph nodes in FVB/C57BL/6J mice. Mesenteric lymph nodes (MLN) from PPAR γ flfl; Villin Cre+ (VC+) or PPAR γ flfl; Villin Cre- (VC-) mice with a mixed FVB/C57BL/6J background (FVB) were immunophenotyped to identify immune cell subsets by flow cytometry. Data were collected on days 0, 2, and 7 of DSS challenge and were analyzed with FACS Diva software. Data are represented as mean ± standard error. Points with an asterisk are significantly different (*P*<0.05).

In the MLN we also observed no significant differences between FVB VC+ and FVB VC- mice in overall percent of immune cells, but there were some distinct phenotypic differences amongst the CD4+ and F4/80+CD11b+ populations (Figure 5). First, the percentage of IL10-expressing CD4+ T cells was higher in the VC- than the VC+ mice. Second, the percent of macrophages expressing MHC II was significantly elevated in the VC+ mice.

**Figure 6:**
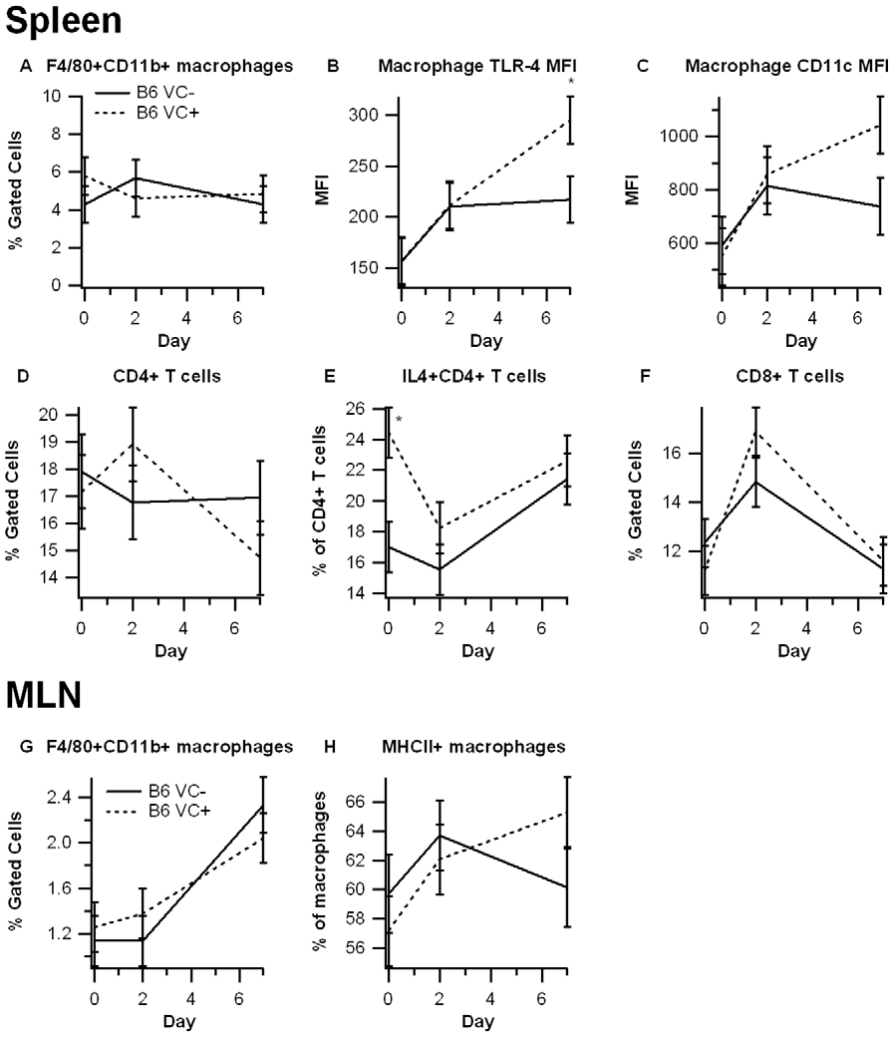
Effect of epithelial cell-specific PPAR γ deletion on immune cell subsets in spleen and mesenteric lymph nodes in C57BL/6J mice. Spleen (A–F) and mesenteric lymph nodes (MLN) (G–H) from PPAR γ flfl; Villin Cre+ (VC+) or PPAR γ flfl; Villin Cre- (VC-) mice with a C57BL/6J background (B6) were immunophenotyped to identify immune cell subsets through flow cytometry. Data were collected on days 0, 2, and 7 of DSS challenge and were analyzed with FACS Diva software. Data are represented as mean ± standard error. Points with an asterisk are significantly different (*P*<0.05).

Similar to the mixed FVB strain, there were no significant differences in F4/80+CD11b+, CD4+, or CD8+ immune cell subsets between B6 VC+ and B6 VC- mice (Figure 6). Interestingly, however, macrophages residing in the spleens of B6 VC+ mice expressed significantly more toll-like receptor-4 (TLR-4) on day 7, and expression of CD11c trended to significance. CD4+ T cells from B6 VC+ mice also expressed significantly more IL-4 on day 0, but this difference was absent on days 2 and 7. Also similar to the FVB strain, MHC II expression was up-regulated in MLN-derived F4/80+CD11b+ macrophages in B6 VC+ mice.

### Global gene expression analysis in the colonic mucosa of VC- and VC+ mice with DSS colitis

Pairwise comparisons revealed very few genes being modulated in the colonic mucosa after 2 days of DSS challenge. In the VC- mice, 1 gene was differentially expressed in the DSS-treated group compared to mice that did not receive DSS. For the same comparison in VC+ mice, 5 genes were shown to have a different expression pattern at day 2 compared to day 0 (Figure S1). However DSS changed the expression of a larger set of 1020 genes at the later time point, day 7. Out of these 1020 genes, 877 genes were differentially expressed in VC- mice only; 38 genes in VC+ mice only and 105 genes differentially expressed in both genotypes (Figure S2).

### Pathways modulated after DSS challenge

Hypergeometric testing of the 877 genes differentially expressed on day 7 of DSS challenge in VC- mice revealed over-representation of six KEGG pathways: Lysosome, DNA Replication, p53 signaling and metabolic pathways (Tables S1, S2, S3, S4, S5, S6, Figures S3, S4, S5, S6, S7). One pathway (lysosome, KEGG Id: 04142) was found to be significantly associated with effect of DSS. For most of the genes examined, gene expression signals were measured to be higher at day 7 compared to day 0 in VC- mice (positive log-fold change in Table S2). Corresponding log-fold changes for VC+ mice were also recorded for comparison purpose and were found to be smaller in magnitude and statistically non-significant. KEGG pathway lysosome (Id: 04142) was obtained and colored according to fold-change of gene expression in VC- mice. Many genes belonging to this pathway are observed to be up-regulated in this pathway (Figure S3). Gene expression in the DNA replication pathway (KEGG Id: 03030) was found to be significantly down-regulated in VC- mice (Table S3, Figure S4). Metabolic (KEGG Ids: 00520, 00240) and signaling (KEGG Id: 04115) pathways were significantly modulated in VC- mice, with heterogeneity regarding direction of differential expression within the same pathway (Tables S4, S5, S6, Figures S5, S6, S7).

### Colonic gene expression by real-time RT-PCR

**Figure 7:**
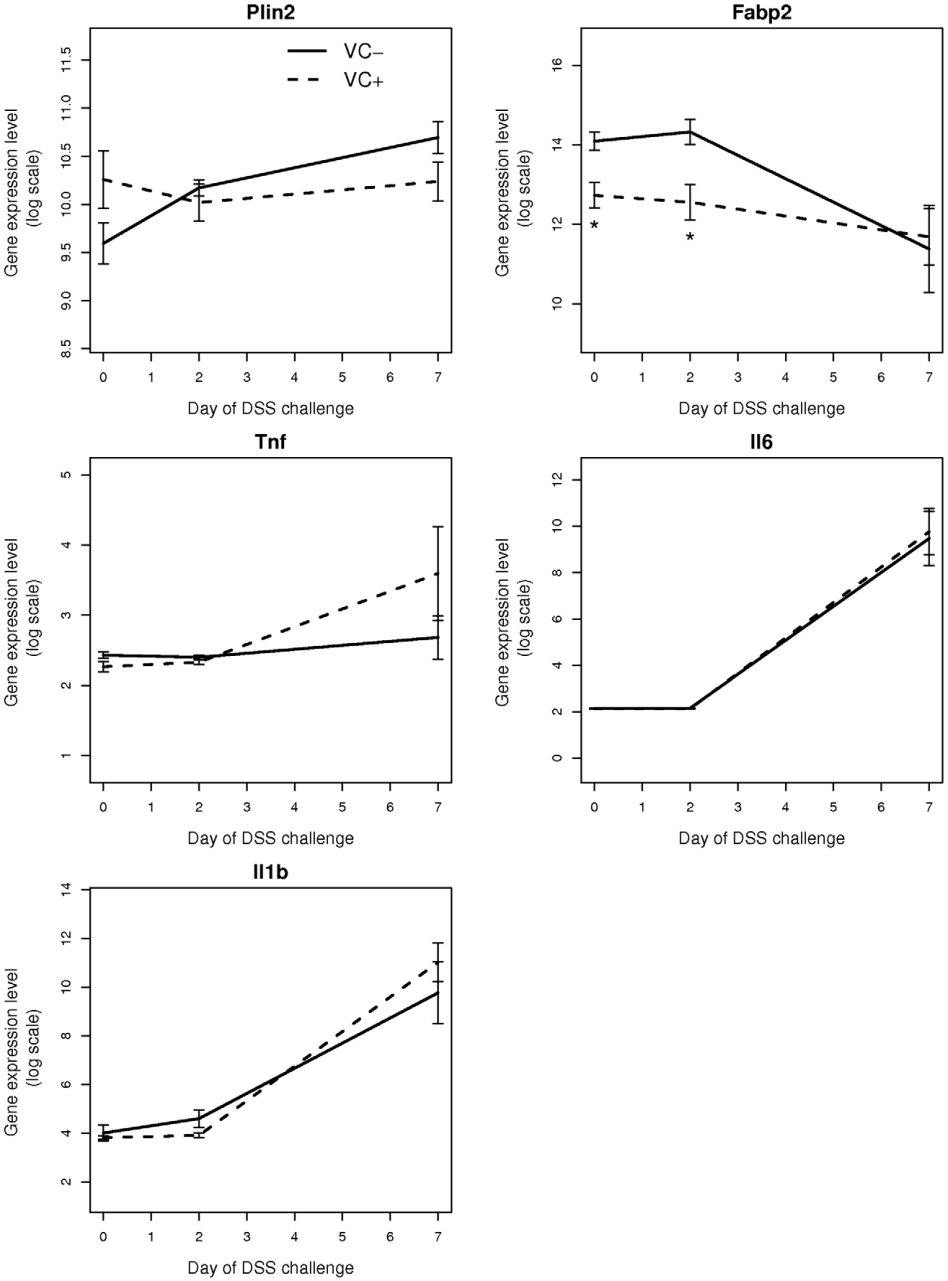
Effect of epithelial cell-specific PPAR γ deletion on target gene expression in colonic mucosa. Expression of PPAR γ targets Plin2 (ADRP), Fabp2 (FABP); and pro-inflammatory genes TNF-α, IL-6 and IL-1β in the colonic mucosa in VC- and VC+ mice at 0, 2 and 7 days after dextran sodium sulfate (DSS) challenge. Compared to VC- mice, VC+ mice show reduced expression of Plin2 (ADRP) at day 7 of DSS challenge and of Fabp2 (FABP) at earlier time points (days 0 and 2). Expression of pro-inflammatory genes TNF-α, IL-6 and IL-1β are increased in VC+ mice compared to VC- mice after DSS challenge. Statistical significance is indicated by asterisks (*P*<0.05).

**Figure 8:**
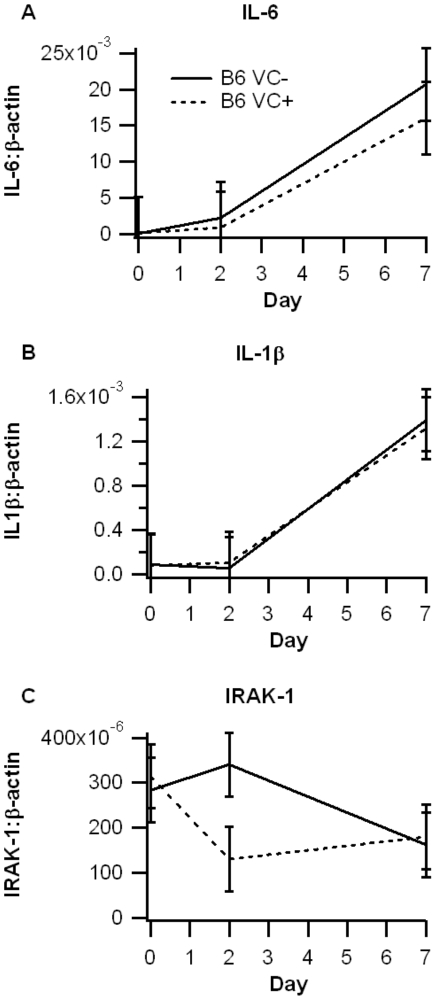
Effect of epithelial cell-specific PPAR γ deletion on target gene expression in colonic mucosa in C57BL/6J mice. Expression of IL-6 (A), IL-1β (B) and IRAK-1 (C) was assessed in the colonic mucosa in PPAR γ flfl; Villin Cre+ (VC+) or PPAR γ flfl; Villin Cre- (VC-) in mice with a C57BL6/J (B6) background. Expression levels were assessed at days 0, 2 and 7 of DSS challenge and normalized relative to the housekeeping gene β-actin. Data are represented as mean ± standard error. There were no statistically significant differences between groups (*P*<0.05).

Adachi et al [12] reported that IEC-specific PPAR γ knock-out mice displayed reduced expression of the PPAR γ target genes Plin2 (ADRP), Fabp2 (FABP) and enhanced expression of pro-inflammatory genes after DSS challenge. We observed the trends in gene expression to be in the same direction as reported by them (Figure 7). Additionally, Fabp2 (FABP) was significantly reduced in VC+ mice compared to VC- mice. Despite showing a significant increase in disease severity, there were no significant differences in the levels of inflammatory proteins IL-1β,IL-6, and IRAK-1, although IRAK-1 expression was numerically down-regulated on day 2 of DSS colitis in IEC-specific PPAR γ null mice (Figure 8).

## Discussion

While PPARs have a well-established role in inflammation [13], the specific contribution of intestinal epithelial cell PPAR γ in IBD is actively under investigation. Adachi et al [12] studied DSS induced colitis in mice with a targeted disruption of PPAR γ function in colonic epithelial cells in a mixed FVB/C57BL6 background. They reported that the lack of endogenous epithelial cell PPAR γ expression in colonic epithelial cells results in increased susceptibility to DSS colitis. Our data demonstrate that there are no differences in clinical parameters of disease between IEC-specific PPAR γ-expressing and null mice in the same interbred genetic background. However, we observe an increased susceptibility to DSS colitis in IEC-specific PPAR γ null mice when the experiment was conducted in a C57BL/6 background, indicating strain-specific differences and suggesting that the Th1- [14] and more pro-inflammatory-prone [15] background may accentuate the role of IEC PPAR γ in regulating mucosal inflammation.

Consistent with the data from Adachi et al. [12], the PPAR γ target Fabp2 (intestinal FABP) is significantly reduced in IEC-specific PPAR γ null mice. The impact of the IEC-specific PPAR γ deletion on pro-inflammatory cytokine gene expression was more modest in our study when compared to Adachi and colleagues. This is also suggested by the lack of clinical (disease activity, body weight) differences between VC- and VC+ mice in a mixed FVB/C57BL/6 background. However, colonic histopathology results reveal significantly greater leukocyte infiltration and epithelial erosion in IEC-specific PPAR γ null mice on day 7 of DSS challenge. After backcrossing the original FVB/C57BL/6 line nine times and generating VC- and VC+ mice in a pure C57BL/6 background the deficiency of PPAR γin IEC resulted in worsened disease activity, greater weight loss and histological differences, suggesting that the Th1-prone genetic background of C57BL/6 mice is optimal for investigating the contribution of IEC PPAR γ to the pathogenesis of IBD. The mice lacking IEC PPAR γ displayed significantly fewer CD4+IL10+ T cells in the MLN and their macrophages expressed greater amounts of TLR4, the molecular target for LPS, in the spleen. Of note, IL-10-producing CD4+ T cells exert regulatory functions, thereby suggesting that IEC PPAR γ is required for the induction of T cell regulatory responses at the mucosal inductive sites and the prevention of experimental IBD.

While histologically IEC-specific PPAR γ null mice demonstrated characteristic colonic inflammatory lesions after DSS challenge, global transcriptome analysis suggests smaller number of genes being altered in IEC-specific PPAR γ null mice in response to DSS in comparison to T cell-specific PPAR γ null mice [11]. More specifically, inflammatory cytokines, adhesion molecules, genes involved in glucose homeostasis, apoptosis and protein synthesis are down-regulated in the colonic mucosa of mice lacking PPAR γ in T cells [11], whereas the lysosomal gene expression represents the primary pathway modulated in IEC-specific PPAR γ null mice. On day 7 of DSS challenge, pathway analysis reveals six pathways to be characteristically associated with DSS in PPAR γ-expressing mice only. The same analysis applied to IEC-specific PPAR γ null mice does not reveal any characteristic pathway to be altered in this phenotype. Because the pathways were selected from the genes that were significantly different between two genotypes on day 7 of DSS challenge (as shown in the Venn Diagram on Figure S2), these pathways are differentially modulated by DSS on day 7.

Genes of the lysosomal pathway are mostly up-regulated in response to DSS in both VC- and VC+ mice (Figure S3, Table S2), although the magnitude of differential expression (fold-change on day 7 compared day 0) is smaller in VC+ mice. A link between IBD and lysosomal alterations have been suggested earlier [16]. More recently, disruption of PPAR γ in mice resulted in focal hyperplasia, accumulation of lysosomes and dysregulation of pathways related to lysosomal maturation in a prostatic cancer model [17], [18]. Cathepsins are lysosomal acid hydrolases whose transcription is enhanced in colonic mucosa in response to dietary DSS. Lysosome associated membrane proteins 1 and 2 (LAMP 1/2) are involved in phagosome maturation in which lysosomes fuse with late phagosomes leading to removal of endocytosed microbes [19]. Gene expression of LAMP 1/2 is increased on day 7 of DSS challenge, with the increase being higher in VC- mice and also statistically significant.

The lack of PPAR γ in IEC interferes with lysosomal gene expression in response to DSS, potentially leading to altered antigen presentation. Presentation of microbe-derived peptides along with MHC class II molecules to the T cell receptor is a central event in induction of antigen-specific CD4^+^ T cell responses. Analysis of the IBD transcriptome in human subjects [20] revealed up-regulation of genes of immune-response and antigen-presentation, although the mechanism by which classical MHC class II genes exert their influence in IBD is currently unknown [21]. IEC are in contact with intra-epithelial lymphocytes (IELs) and equipped with the machinery for antigen processing and presentation [22]. On the other hand IEC PPAR γ serves an important role in suppressing pro-inflammatory cytokine expression and represents a molecular target of anti-inflammatory commensal bacteria [23]. Since lysosomal degradation represents an essential step in antigen presentation via MHC class II, the finding that the targeted disruption of IEC PPAR γ results in altered expression of lysosomal pathway genes may be indicative of a possible role of IEC PPAR γ in the induction of CD4+ T cell regulatory responses by increasing presentation of commensal bacterial antigens.

In summary, by using an IEC-targeted loss-of-function approach we show that expression of IEC PPAR γ is required for preventing colonic inflammatory lesions, up-regulating lysosomal pathway genes and increasing the production of the anti-inflammatory cytokine, IL-10, by CD4+ T cells in the MLN of mice with experimental IBD.

## Materials and Methods

### Ethics statement

All experimental procedures were approved by the Institutional Animal Care and Use Committee (IACUC) of Virginia Polytechnic Institute and State University (IACUC approval number 08-082-VBI) and met or exceeded requirements of the Public Health Service/National Institutes of Health and the Animal Welfare Act.

### Mouse Genotyping

Intestinal tissue specimens and isolated intestinal epithelial cells were obtained from tissue-specific PPAR γ fl/fl; Villin Cre+ (VC+) and Villin Cre- control mice (VC-) kindly provided by Dr. Frank Gonzalez (NCI, Bethesda, MD). Duodenum, jejunum, ileum, cecum and colon were excised. For cell isolation, the different parts of the intestine were washed with PBS, minced and incubated twice in CMF/FBS/EDTA media at 37°C for 15 minutes. The digest was passed through a nylon mesh and supernatants were collected and centrifuged. Cells were washed and resuspended in lysis buffer. DNA was extracted by using the QIAamp DNA Mini Kit, Blood and Body Fluid Spin Protocol (Qiagen). For whole tissue DNA isolation, each part of the intestine was collected and homogenized and then genomic DNA was extracted by QIAamp DNA Mini Kit, Tissue Protocol (Qiagen). PCR was performed as previously described [8], [12] and PCR amplifications were resolved through ethidium bromide staining on a 2% agarose gel and run at 120 V for 30 minutes. No differences in the expression of the Villin-Cre recombinase or recombination efficiency were found between the intestines of VC+ mice in a mixed FVB/C57BL/6 and the CB57BL/6 backgrounds (data not shown).

### Animal Procedures

PPAR γ flfl; Villin Cre^+^ (FVB VC+, n = 45) and control PPAR γ flfl; Villin Cre^-^ (FVB VC-, n = 42) mice in a mixed FVB/C57BL/6 background [12] were used for these experiments. These mice express a transgenic recombinase under the control of the Villin-Cre promoter. To facilitate more meaningful comparisons with other mouse knockout strains, we backcrossed these mice nine times to a C57BL/6 background and generated B6 VC- (n = 15) and B6 VC+ (n = 15) mice in a C57BL/6 background. The mice were housed at the animal facilities at Virginia Polytechnic Institute and State University in a room maintained at 75° F, with a 12∶12 h light-dark cycle starting from 6:00 AM. Mice were challenged with 2.5% dextran sodium sulfate (DSS), 36,000–44,000 molecular weight (ICN Biomedicals, Aurora, OH) in the drinking water. DSS damages the epithelial barrier leading to colitis with involvement of macrophages and later, T cells [24]. After the DSS challenge mice were weighed on a daily basis and examined for clinical signs of disease associated with colitis (i.e., perianal soiling, rectal bleeding, diarrhea, and piloerection). For the DSS challenge, the disease activity indices and rectal bleeding scores were calculated using a modification of a previously published compounded clinical score. Briefly, disease activity index consisted of a scoring for diarrhea and lethargy (0–3), whereas rectal bleeding consisted of a visual observation of blood in feces and the perianal area (0–4). Mice in the DSS study were euthanized on days 0, 2, and 7 of the DSS challenge by carbon dioxide narcosis followed by secondary thoracotomy and blood was withdrawn from the heart. Spleen and mesenteric lymph nodes (MLN) were scored based on size and macroscopic inflammatory lesions (0–3), excised, and single-cell suspensions were prepared as previously described for flow cytometry [3].

### Histopathology

Colonic sections were fixed in 10% buffered neutral formalin, later embedded in paraffin, and then sectioned (5 µm) and stained with hematoxylin and eosin (H&E) for examination of microscopic lesions and changes in the mucosal architecture. Colons were graded with a compounded histologic score including the extent of (1) leukocyte infiltration, (2) mucosal thickening, and (3) epithelial cell erosion. The sections were graded with a score of 0–4 for each of the previous categories and data were analyzed as a normalized compounded score.

### Immunophenoptying of blood, spleen, and MLN

MLN and spleen-derived cells or whole blood were seeded onto 96-well plates, centrifuged at 4°C at 3000 rpm for 4 minutes, and washed with PBS containing 5% serum and 0.09% sodium azide (FACS buffer). To assess differential monocyte/macrophage subsets, the cells were then incubated in the dark at 4°C for 20 minutes in FcBlock (20 µg/ml; BD Pharmingen), and then for an additional 20 minutes with fluorochrome-conjugated primary antibodies anti-F4/80-PE-Cy5 (5 µg/mL, ebioscience), anti-CD11b-Alexa Fluor 700 (2 µg/mL, eBioscience) and anti-MHC II-PE (2 µg/mL, eBioscience). For lymphocyte subset assessment, cells were incubated with anti-CD4-Alexa Fluor 700 (2 µg/mL; BD Pharmingen), anti-CD8-PerCp-Cy5.5 (2 µg/mL, eBioscience), CD3 PE-Cy5 (2 µg/mL; BD Pharmingen), anti-FoxP3-PE (2 µg/mL, eBioscience), and anti-IL10-PE as previously shown [3]. Flow results were computed with a BD LSR II flow cytometer and data analyses was performed with FACS Diva software (BD).

### Microarray data analysis

After homogenization of colonic tissue, total RNA was extracted and purified using the RNAeasy system according to manufacturer's instructions (Qiagen Valencia, CA). The QIAGEN RNase-free DNase supplement kit was used to ensure that the RNA was free from DNA contamination. RNA was then processed and labeled according to the standard target labeling protocols and the samples were hybridized, stained, and scanned per standard Affymetrix protocols at VBI core laboratory on Mouse 430 2.0 expression arrays (Affymetrix Inc., Santa Clara, CA). All statistical analysis of the data was performed within R statistical environment - Version 2.10.1 [25] using Bioconductor packages. Raw microarray data from CEL files were read with ‘affy’ package [26] and pre-processed by GC-RMA algorithm that performs the three steps: (i) adjustment of the gene expression signal against the background caused by optical noise and non-specific binding, (ii) robust multi-array normalization, and (iii) summarization of the probes belonging to each probe set. At the outset, a non-specific filter using the function nsFilter from bioconductor package ‘genefilter’ was applied to remove non-informative probe sets that displayed low variance across all samples. Differential expression analysis was performed using the package limma [27]. A linear model was fit, using the function lmFit, to the expression data (log-intensities) for each gene. The fitted coefficients were compared (DSS versus No DSS; at two time points, day 2 and day 7) using the function contrasts.fit. Empirical Bayes method [28], [29] was used to borrow information across genes. This has been shown to make the analysis stable for experiments with small number of arrays. P-values obtained for each gene was corrected for multiple comparisons [30] and a cutoff of 0.05 was applied to identify the genes that are significantly differentially expressed between the conditions. Venn Diagrams were drawn using the function vennDiagram to show the number of genes differentially expressed under different conditions. The microarray data (both raw and normalized) have been submitted at the Gene Expression Omnibus (GEO, http://www.ncbi.nlm.nih.gov/geo/, Series: GSE20621).

### Hypergeometric testing for over-represented pathways

All pathways listed at Kyoto Encylopedia for Genes and Genomes (KEGG) were selected for analysis. Genes that were differentially expressed after 7 days of DSS challenge were subjected to hypergeometric testing, with the function hyperGTest from Category package, for discovery of the over-represented KEGG pathways. This procedure used Fisher's exact test to find association between interesting genes (differentially expressed after 7 days of DSS challenge) and membership to a KEGG pathway. Selected KEGG pathways found to be significantly associated with DSS on day 7 were accessed using the bioconductor package KEGGSOAP. Specific gene nodes on each pathway were ‘painted’ according to the direction of differential expression of that gene on day 7 of DSS treatment: red if up-regulated, green if down-regulated.

### Quantitative Real-Time RT-PCR

Total RNA (1 µg) from colons was used to generate a complementary DNA (cDNA) template using the iScript cDNA Synthesis Kit (Bio-Rad, Hercules, CA) using previously described conditions [8]. Each gene amplicon was purified with the MiniElute PCR Purification Kit (Qiagen) and quantitated on an agarose gel by using a DNA mass ladder (Promega). These purified amplicons were used to optimize real-time PCR conditions and to generate standard curves in the real-time PCR assay. Primer concentrations and annealing temperatures were optimized for the iCycler iQ system (Bio-Rad) for each set of primers using the system's gradient protocol. PCR efficiencies were maintained between 92 and 105% and correlation coefficients above 0.98 for each primer set during optimization and also during the real-time PCR of sample DNA.

Complementary DNA (cDNA) concentrations for genes of interest were examined by real-time quantitative PCR using an iCycler IQ System and the iQ SYBR green supermix (Bio-Rad). A standard curve was generated for each gene using 10-fold dilutions of purified amplicons starting at 5 pg of cDNA and used later to calculate the starting amount of target cDNA in the unknown samples. SYBR green I is a general double-stranded DNA intercalating dye and may therefore detect nonspecific products and primer/dimers in addition to the amplicon of interest. In order to determine the number of products synthesized during the real-time PCR, a melting curve analysis was performed on each product. Real-time PCR was used to measure the starting amount of nucleic acid of each unknown sample of cDNA on the same 96-well plate. Genebank accession numbers used for the forward and reverse primers are as follows: β-actin (X03672) forward 5′CCCAGGCATTGCTGACAGG3′ and reverse5′TGGAAGGTGGACAGTGAGGC3′ ; IL-6 (NM_031168) forward 5′TTTCCTCTGGTCTTCTGGAG3′ and reverse 5′CTGAAGGACTCTGGCTTTGT3′; IL-1β (NM_008361) forward GGGTCGGACTGTTTCTAAGTC3′ and reverse 5′CTTGGCCGAGGACTAAG3′; IRAK-1 (NM_008363) forward 5′CGCCAAGCACTTCTTGTACGA′3 and reverse 5′GATCAAGGCCGCGAACT3′.

### Statistics

Flow cytometry, disease activity, pathology and real-time RT-PCR data were analyzed as a repeated measures 3×2 factorial arrangement within a completely randomized design. To determine the statistical significance of the model, analysis of variance (ANOVA) was performed using the general linear model procedure of Statistical Analysis Software (SAS), and probability value (*P*) <0.05 was considered to be significant. When the model was significant, ANOVA was followed by Fisher's Protected Least Significant Difference multiple comparison method.

## Supporting Information

Figure S1 Venn diagram showing number of genes differentially expressed on day 2 of Dextran Sodium Sulfate (DSS) challenge. The number inside each circle refers to number of genes differentially expressed on 2nd day of DSS challenge (compared to control, i.e., day 0), for each genotype VC-, VC+. The number inside overlapping region of two circles refers to the number of genes that are common to both genotypes. The number on the bottom right corner corresponds to genes that are not differentially expressed.
(0.05 MB TIF)

Figure S2 Venn diagram showing number of genes differentially expressed on day 7 of Dextran Sodium Sulfate (DSS) challenge. The number inside each circle refers to number of genes differentially expressed on 7th day of DSS challenge (compared to control, i.e., day 0), for each genotype VC-, VC+. The number inside overlapping region of two circles refers to the number of genes that are common to both genotypes. The number on the bottom right corner corresponds to genes that are not differentially expressed.
(0.05 MB TIF)

Figure S3 Lysosomal genes differentially expressed on day 7 of DSS challenge in VC- mice. The pathway diagram from KEGG has been colored according to the direction of change in gene expression. Significantly up-regulated genes are colored in red.
(0.59 MB TIF)

Figure S4 DNA replication (KEGG) genes differentially expressed on day 7 of DSS challenge in VC- mice. The pathway diagram from KEGG has been colored according to the direction of change in gene expression. Significantly down-regulated genes on day 7 in VC- mice are colored in green.
(0.62 MB TIF)

Figure S5 Genes of the KEGG pathway "Aminosugar and nucleotide sugar metabolism" are differentially expressed on day 7 of DSS challenge in VC- mice. The pathway diagram from KEGG has been colored according to the direction of change in gene expression. Significantly up-regulated genes on day 7 in VC- mice are colored in red; down-regulated in green.
(0.65 MB TIF)

Figure S6 Genes of the KEGG pathway "Pyrimidine metabolism" are differentially expressed on day 7 of DSS challenge in VC- mice. The pathway diagram from KEGG has been colored according to the direction of change in gene expression. Significantly down-regulated genes on day 7 in VC- mice are colored in green; up-regulated in red.
(0.57 MB TIF)

Figure S7 Genes of the KEGG "p53 Signaling Pathway" are differentially expressed on day 7 of DSS challenge in VC- mice. The pathway diagram from KEGG has been colored according to the direction of change in gene expression. Significantly up-regulated genes on day 7 in VC- mice are colored in red; down-regulated in green.
(0.31 MB TIF)

Table S1 KEGG pathways modulated on day 7 of DSS challenge in VC- mice. A total of 877 genes, transcriptionally affected on day 7 of VC- (but not VC+) mice, were subjected to hypergeometric testing and revealed enrichment (over-representation) of six KEGG pathways. The first and last columns correspond to the KEGG identifier and name of the pathway, respectively. The second and third columns (Pvalue, OddsRatio) report that there is good association between DSS challenge and the KEGG pathway in VC- mice. The ExpCount records the expected number of genes in the selected gene list to be found at the KEGG pathway, which is exceeded by the actual Count (fifth column). Sixth column (Size) corresponds to the total number of genes in the pathway.
(0.01 MB XLS)

Table S2 Lysosomal genes differentially expressed on day 7 of DSS challenge in VC- mice. Log fold-change in gene expression on day 7 after DSS challenge in VC- and VC+ mice. Degree of differential expression of these genes is statistically significant for VC- mice (suggested by asterisk). Corresponding fold changes in VC+ mice are not statistically significant. Most of the genes are up-regulated in VC- mice.
(0.02 MB XLS)

Table S3 DNA replication (KEGG) genes differentially expressed on day 7 of DSS challenge in VC- mice. Log fold-change in gene expression on day 7 after DSS challenge in VC- and VC+ mice. Degree of differential expression of these genes is statistically significant for VC- mice (suggested by asterisk). Corresponding fold changes in VC+ mice are not statistically significant. The genes are down-regulated in VC- mice.
(0.02 MB XLS)

Table S4 Genes of the KEGG pathway "Aminosugar and nucleotide sugar metabolism" are differentially expressed on day 7 of DSS challenge in VC- mice. Log fold-change in gene expression on day 7 after DSS challenge in VC- and VC+ mice. Degree of differential expression of these genes is statistically significant for VC- mice (suggested by asterisk). Corresponding fold changes in VC+ mice are not statistically significant.
(0.02 MB XLS)

Table S5 Genes of the KEGG pathway "Pyrimidine metabolism" are differentially expressed on day 7 of DSS challenge in VC- mice. Log fold-change in gene expression on day 7 after DSS challenge in VC- and VC+ mice. Degree of differential expression of these genes is statistically significant for VC- mice (suggested by asterisk). Corresponding fold changes in VC+ mice are not statistically significant. The genes are mostly down-regulated in VC- mice.
(0.02 MB XLS)

Table S6 Genes of the KEGG "p53 Signaling Pathway" are differentially expressed on day 7 of DSS challenge in VC- mice. Log fold-change in gene expression on day 7 after DSS challenge in VC- and VC+ mice. Degree of differential expression of these genes is statistically significant for VC- mice (suggested by asterisk). Corresponding fold changes in VC+ mice are not statistically significant.
(0.02 MB XLS)
