# Supplementary figures and images for: Immunoregulatory Actions of Epithelial Cell PPAR γ at the Colonic Mucosa of Mice with Experimental Inflammatory Bowel Disease
Source: PLoS One. 2010 Apr 20;5(4):e10215. doi: 10.1371/journal.pone.0010215 (PMC2857885; doi:10.1371/journal.pone.0010215)

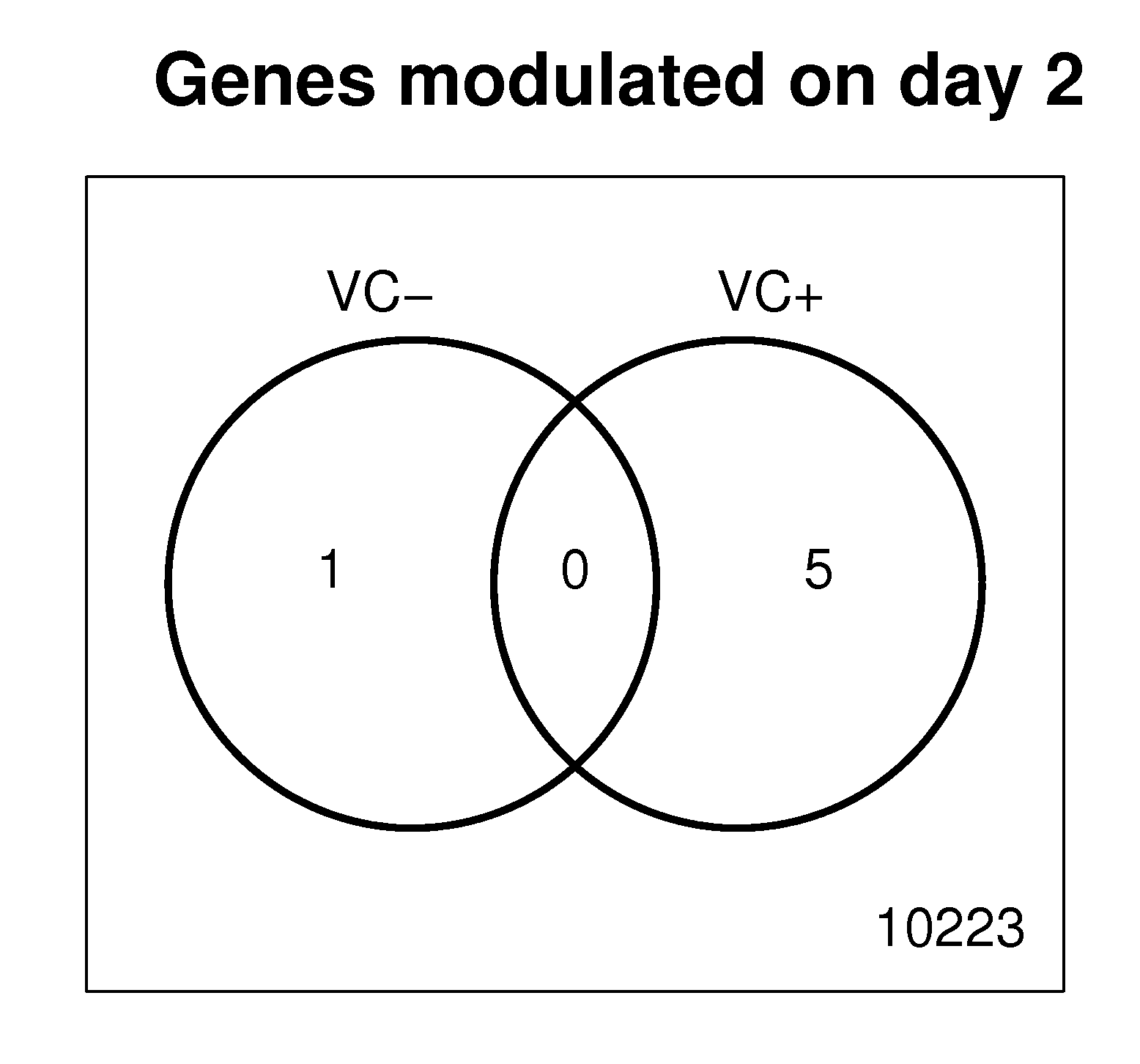

Supplement: Figure S1 — Venn diagram showing number of genes differentially expressed on day 2 of Dextran Sodium Sulfate (DSS) challenge. The number inside each circle refers to number of genes differentially expressed on 2nd day of DSS challenge (compared to control, i.e., day 0), for each genotype VC-, VC+. The number inside overlapping region of two circles refers to the number of genes that are common to both genotypes. The number on the bottom right corner corresponds to genes that are not differentially expressed. (0.05 MB TIF) [file pone.0010215.s001.tif]

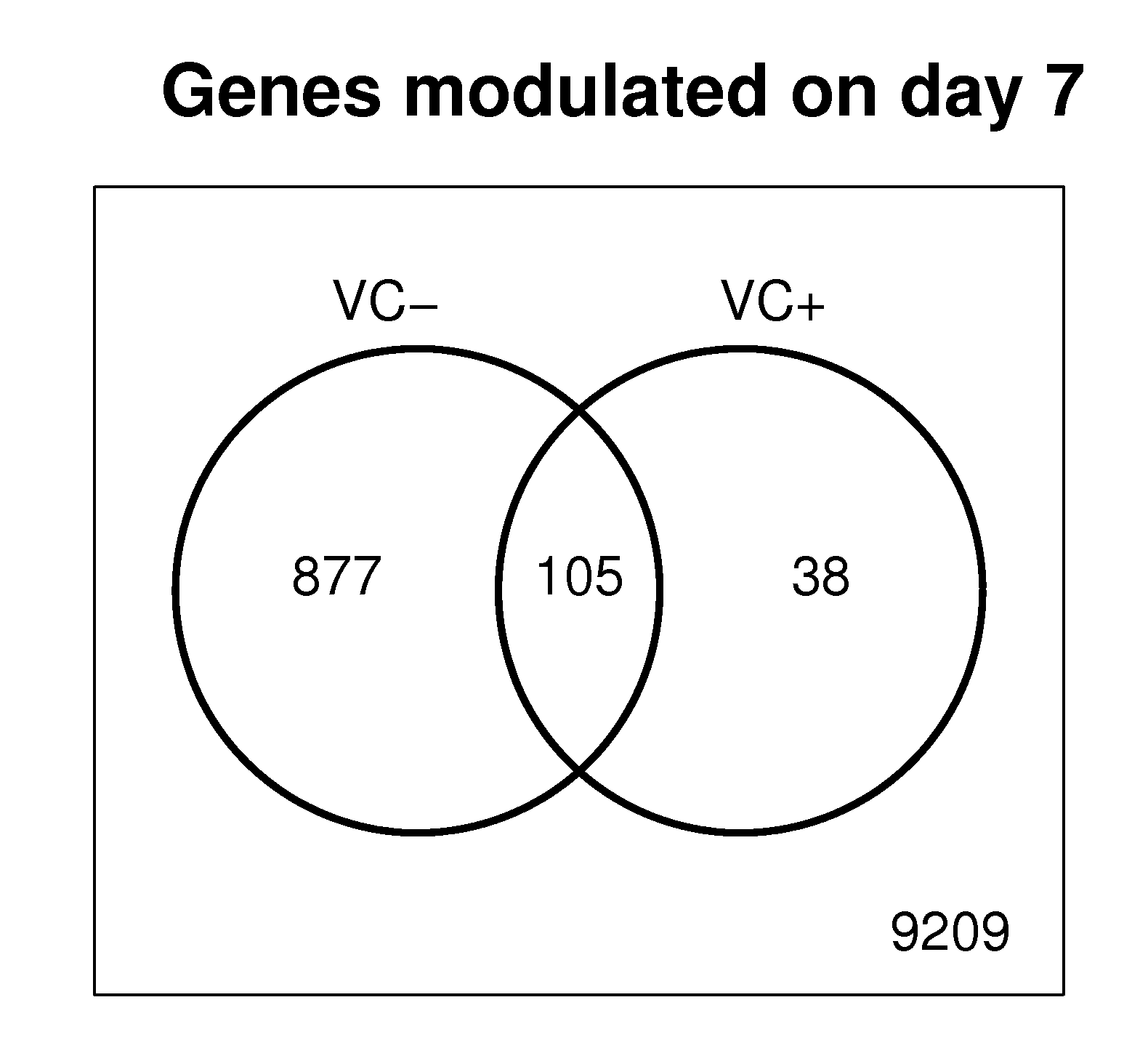

Supplement: Figure S2 — Venn diagram showing number of genes differentially expressed on day 7 of Dextran Sodium Sulfate (DSS) challenge. The number inside each circle refers to number of genes differentially expressed on 7th day of DSS challenge (compared to control, i.e., day 0), for each genotype VC-, VC+. The number inside overlapping region of two circles refers to the number of genes that are common to both genotypes. The number on the bottom right corner corresponds to genes that are not differentially expressed. (0.05 MB TIF) [file pone.0010215.s002.tif]

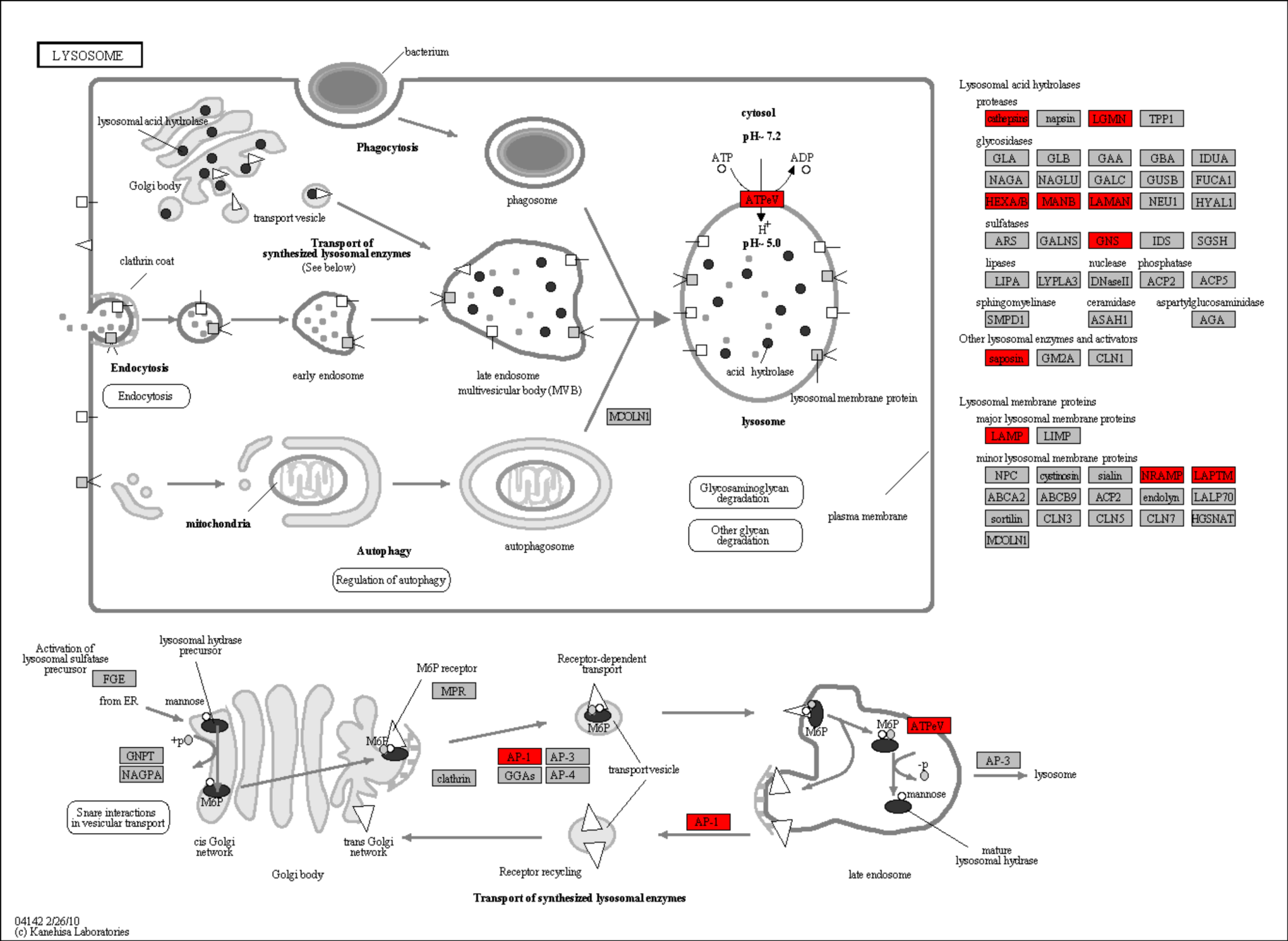

Supplement: Figure S3 — Lysosomal genes differentially expressed on day 7 of DSS challenge in VC- mice. The pathway diagram from KEGG has been colored according to the direction of change in gene expression. Significantly up-regulated genes are colored in red. (0.59 MB TIF) [file pone.0010215.s003.tif]

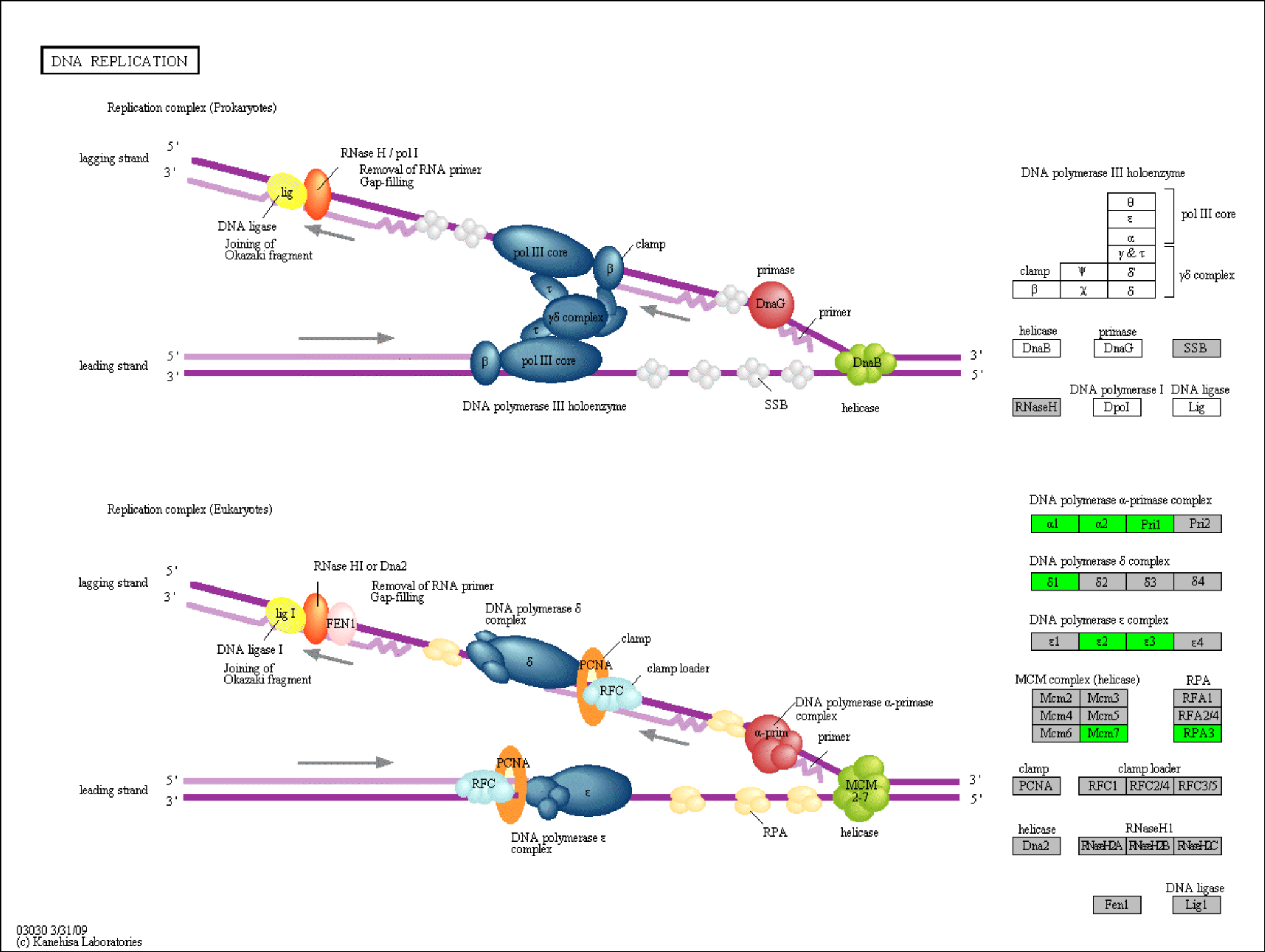

Supplement: Figure S4 — DNA replication (KEGG) genes differentially expressed on day 7 of DSS challenge in VC- mice. The pathway diagram from KEGG has been colored according to the direction of change in gene expression. Significantly down-regulated genes on day 7 in VC- mice are colored in green. (0.62 MB TIF) [file pone.0010215.s004.tif]

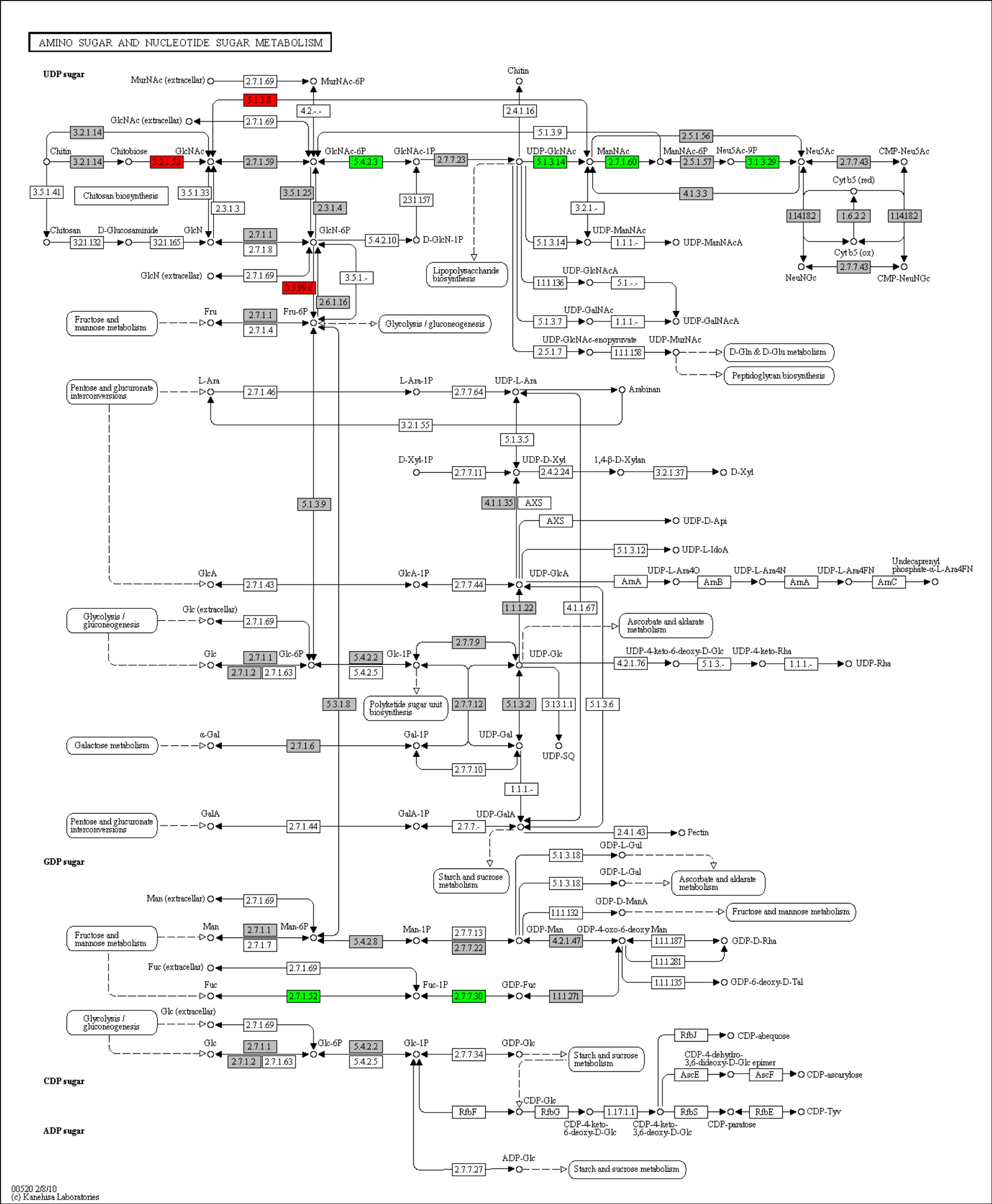

Supplement: Figure S5 — Genes of the KEGG pathway "Aminosugar and nucleotide sugar metabolism" are differentially expressed on day 7 of DSS challenge in VC- mice. The pathway diagram from KEGG has been colored according to the direction of change in gene expression. Significantly up-regulated genes on day 7 in VC- mice are colored in red; down-regulated in green. (0.65 MB TIF) [file pone.0010215.s005.tif]

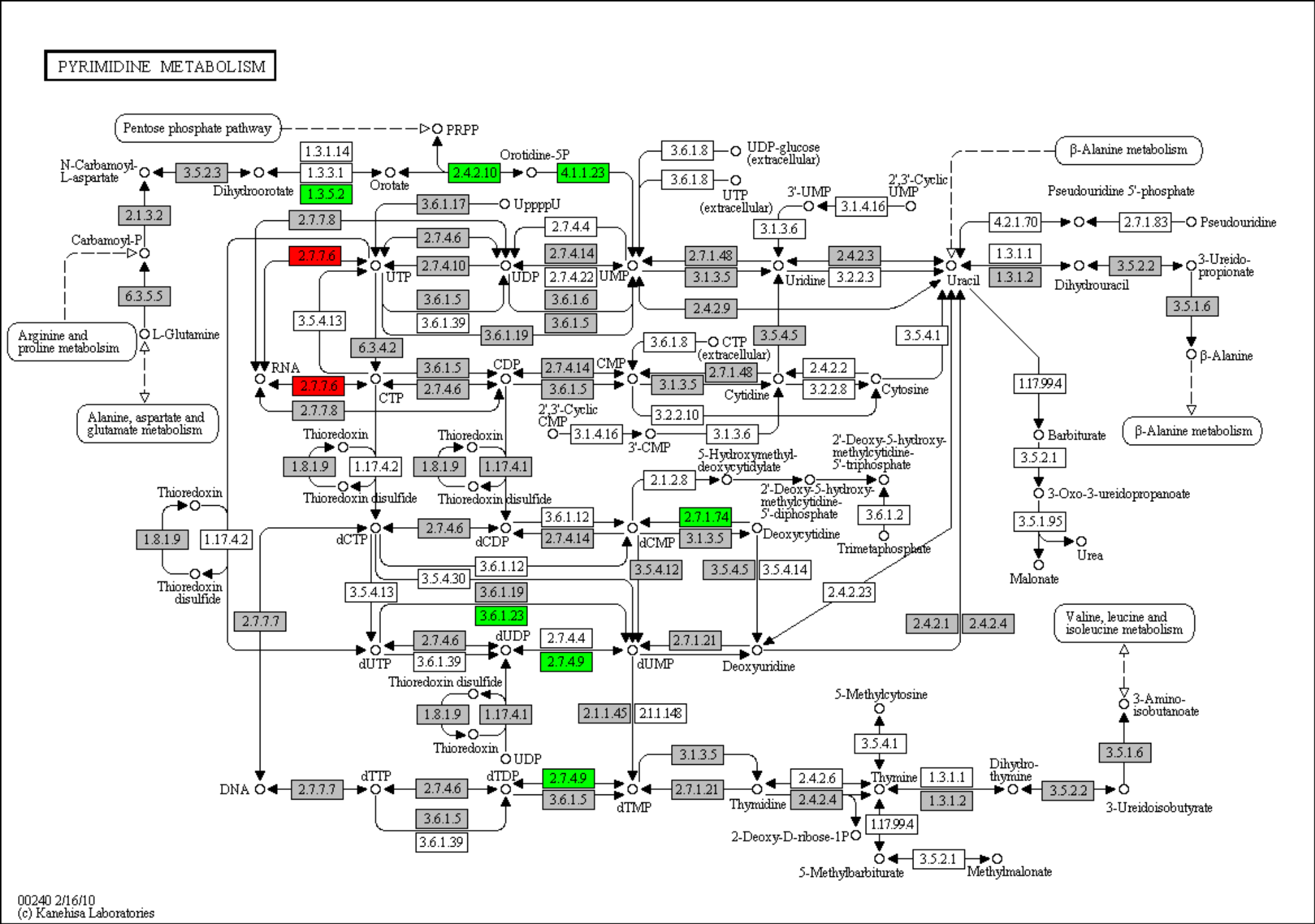

Supplement: Figure S6 — Genes of the KEGG pathway "Pyrimidine metabolism" are differentially expressed on day 7 of DSS challenge in VC- mice. The pathway diagram from KEGG has been colored according to the direction of change in gene expression. Significantly down-regulated genes on day 7 in VC- mice are colored in green; up-regulated in red. (0.57 MB TIF) [file pone.0010215.s006.tif]

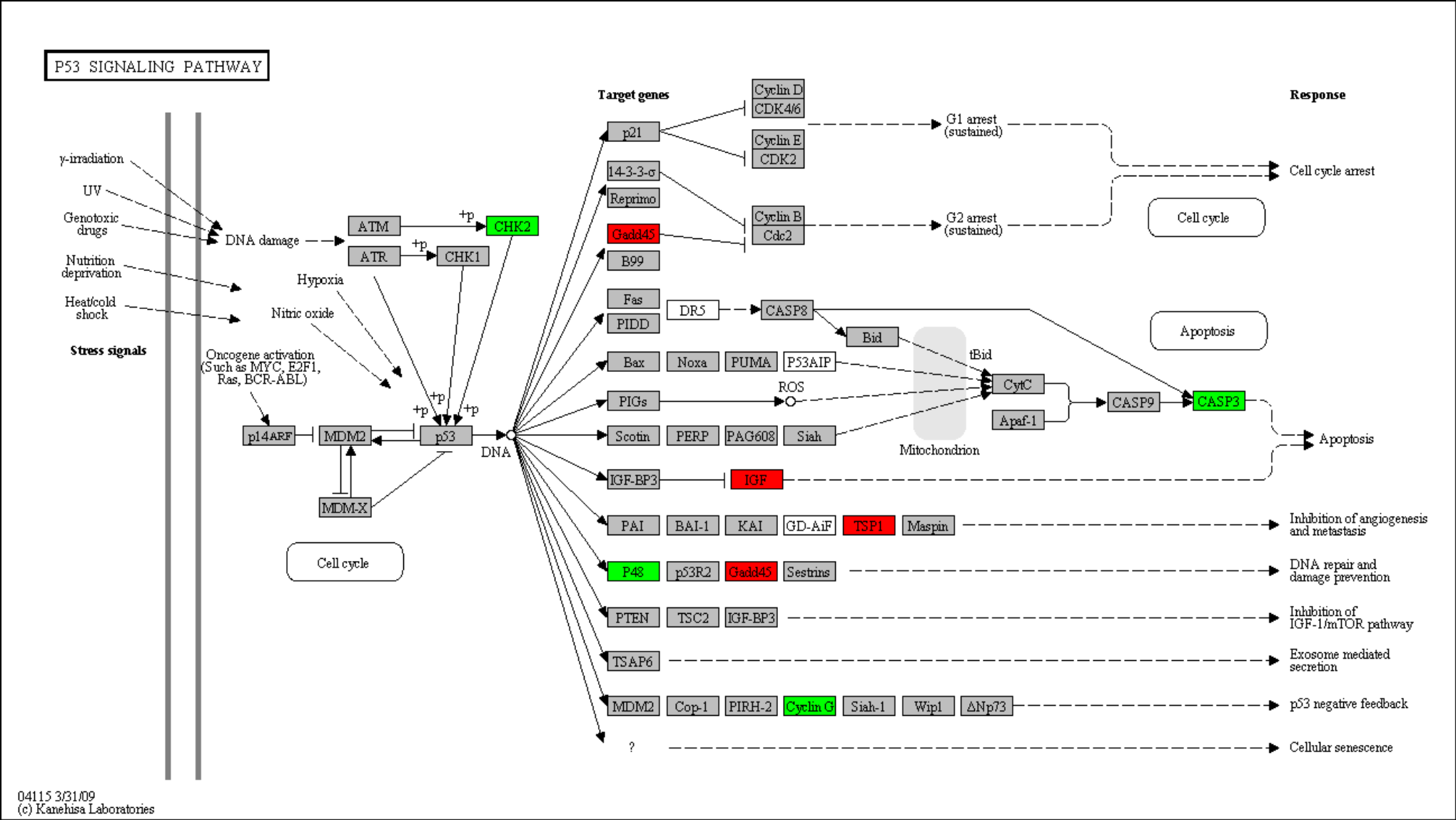

Supplement: Figure S7 — Genes of the KEGG "p53 Signaling Pathway" are differentially expressed on day 7 of DSS challenge in VC- mice. The pathway diagram from KEGG has been colored according to the direction of change in gene expression. Significantly up-regulated genes on day 7 in VC- mice are colored in red; down-regulated in green. (0.31 MB TIF) [file pone.0010215.s007.tif]
